# G Protein-Coupled Estrogen Receptor Protects From Angiotensin II-Induced Increases in Pulse Pressure and Oxidative Stress

**DOI:** 10.3389/fendo.2019.00586

**Published:** 2019-08-27

**Authors:** Benard O. Ogola, Margaret A. Zimmerman, Venkata N. Sure, Kaylee M. Gentry, Jennifer L. Duong, Gabrielle L. Clark, Kristin S. Miller, Prasad V. G. Katakam, Sarah H. Lindsey

**Affiliations:** ^1^Department of Pharmacology, Tulane University, New Orleans, LA, United States; ^2^Department of Biomedical Engineering, Tulane University, New Orleans, LA, United States

**Keywords:** estrogen, G protein-coupled estrogen receptor, NADPH oxidase 4, oxidative stress, cell signaling/signal transduction

## Abstract

Our previous work showed that the G protein-coupled estrogen receptor (GPER) is protective in the vasculature and kidneys during angiotensin (Ang) II-dependent hypertension by inhibiting oxidative stress. The goal of the current study was to assess the impact of GPER deletion on sex differences in Ang II-induced hypertension and oxidative stress. Male and female wildtype and GPER knockout mice were implanted with radiotelemetry probes for measurement of baseline blood pressure before infusion of Ang II (700 ng/kg/min) for 2 weeks. Mean arterial pressure was increased to the same extent in all groups, but female wildtype mice were protected from Ang II-induced increases in pulse pressure, aortic wall thickness, and Nox4 mRNA. *In vitro* studies using vascular smooth muscle cells found that pre-treatment with the GPER agonist G-1 inhibited Ang II-induced ROS and NADP/NADPH. Ang II increased while G-1 decreased Nox4 mRNA and protein. The effects of Ang II were blocked by losartan and Nox4 siRNA, while the effects of G-1 were inhibited by adenylyl cyclase inhibition and mimicked by phosphodiesterase inhibition. We conclude that during conditions of elevated Ang II, GPER via the cAMP pathway suppresses Nox4 transcription to limit ROS production and prevent arterial stiffening. Taken together with our previous work, this study provides insight into how acute estrogen signaling via GPER provides cardiovascular protection during Ang II hypertension and potentially other diseases characterized by increased oxidative stress.

## Introduction

Premenopausal women are protected from cardiovascular disease compared with age-matched men, while aging narrows this sex difference (1). The G protein-coupled estrogen receptor (GPER), previously known as GPR30, mediates non-genomic signaling by estrogen and is expressed in vascular endothelial and smooth muscle cells (2–4). Numerous ligands bind to GPER such as estradiol (3), 2-methoxyestradiol (5), genistein (6), and the selective agonist G-1 (7). We previously showed that G-1 induces vasorelaxation by inducing nitric oxide release from endothelial cells and increasing cAMP signaling in smooth muscle cells (8). Pharmacological activation of GPER ameliorates maladaptive tissue remodeling in the vasculature, heart, and kidneys of salt-sensitive mRen2 female rats (9–11), as well in doxorubicin-induced cardiotoxicity (12). Global GPER deletion does not impact reproductive function yet induces a variety of cardiometabolic deficits (13) including increased fat mass (14, 15), atherosclerosis (16), blood pressure, and glucose intolerance (17). Moreover, the first study to conditionally delete GPER shows that cardiomyocyte GPER is important for cardiac structure and function in both sexes (18). Interestingly, while GPER is expressed in the vasculature of both sexes (19, 20), the protective effects of GPER seem to be reversed in aging male mice, where global GPER deletion is protective against cardiac and vascular dysfunction (21, 22). Many of the cardiovascular effects of GPER are associated with changes in reactive oxygen species, suggesting an antioxidant role for this estrogen receptor.

Reactive oxygen species (ROS) are free radical and non-radical oxygen species including superoxide (${\text{O}}_{2}^{-}$), hydrogen peroxide (H_2_O_2_), peroxynitrite (${\text{NO}}_{3}^{-}$), and hydroxyl radical (HO•). Excessive ROS overwhelms the cellular antioxidant system, causes oxidative stress, and promotes atherosclerosis, hypertension, stroke, and pathophysiological vascular remodeling (23–26). NADPH oxidase (Nox) proteins mediate electron transfer through catalytic subunits and significantly contribute to the production of ROS including superoxide and H_2_O_2_ (27). The Nox family of enzymes consists of Nox1 to Nox5 and dual oxidases (Duox) Duox1 and Duox2 which play diverse roles in the cardiovascular system (28). Many of the deleterious effects of Ang II are attributed to the generation of ROS via the recruitment of Nox proteins, as well as accessory proteins, to form a complex at the membrane (29). More specifically, Ang II upregulates the expression of Nox4 in vascular smooth muscle cells (30), an effect that is reversed by increasing cAMP signaling (31). Increased Nox4 expression is also found in models of Ang II-dependent hypertension including the spontaneously hypertensive rat (31) and the mRen2 rodent model (30). Therefore, the regulation of Nox proteins may be critical in mediating the adverse effects of Ang II.

Since GPER decreases NADPH-generated superoxide in carotid and intracranial arteries (32), Nox proteins may play a role in its vascular antioxidant effects. *In vitro* application of a GPER antagonist upregulates Nox1 but not Nox2 or Nox4, while global GPER deletion is associated with lower expression of Nox1 in the aorta and heart of aging male mice (21). In contrast to the lack of changes in Nox4 in male mice, ovariectomy-induced upregulation of cardiac Nox4 is prevented by chronic administration of the GPER agonist G-1 (11), while cardiomyocyte-specific GPER deletion in female mice induces a 4-fold increase in Nox4 mRNA (33). Therefore, the objective of this study was to investigate sex differences in the impact of GPER on Ang II-induced hypertension, oxidative stress, and Nox expression. We hypothesized that female responses to Ang II would be lower than males, while global GPER deletion would attenuate the protective effects of female sex. Moreover, we hypothesized that the antioxidant effects of GPER would be associated with changes in Nox.

## Materials and Methods

### Animals

All procedures were carried out in accordance with the NIH Guide for the Care and Use of Laboratory Animals and approved by the Tulane University Institutional Animal Care and Use Committee. The GPER knockout strain used in this study was derived from the original model created by homologous recombination (17, 34). Male and female wildtype and global GPER knockout mice were bred and maintained in the institutional vivarium. The presence or absence of GPER was verified using both genotyping and ddPCR as previously described (35). Mice had free access to food and water in a temperature-controlled room (65–75°F) with a 12 h light to dark cycle. Mice were anesthetized for implantation of radiotelemetry probes in the carotid artery. After recovery and recording of baseline cardiovascular parameters, osmotic minipumps (Alzet Model 1002) containing Ang II (Bachem) were implanted to infuse at a rate of 700 ng/kg/min for 2 weeks, a protocol previously shown to induce sex differences in Ang II-induced hypertension (36, 37). Mice were euthanized at 18–25 weeks of age using isoflurane, and mesenteric arteries were harvested for measurement of vascular reactivity as described below. Aortas were stripped of fat, washed in PBS, and stored in −80°C until use. Male and female Sprague Dawley rats were obtained at 3–6 months of age from Charles River for use in cell culture studies.

### Vascular Reactivity

Mesenteric arteries were cleaned of surrounding connective tissue, cut into 2-mm ring segments, and mounted on two wires connected to an isometric force transducer (DMT 620 M, Ann Arbor, MI). Segments were bathed in Krebs buffer (118 mM NaCl, 25 mM NaHCO_3_, 4.8 mM KCl, 2.5 mM CaCl_2_, 1.2 mM MgSO_4_, 1.2 mM KH_2_PO_4_, and 11 mM glucose; pH 7.4) and mixed with 95% O_2_ and 5% CO_2_ at 37°C. Normalization and assessment of baseline vascular dynamics were done as previously described (35). Vascular contractility was assessed in response to increasing concentrations of angiotensin II (Ang II; 10^−10^ to 10^−6^ M) and prostaglandin F2α (PGF2α; 10^−8^ to 10^−4^ M). Vascular relaxation to increasing concentrations of sodium nitroprusside (SNP) or acetylcholine (10^−10^ to 10^−5^ M) were assessed in vessels pre-constricted with 10^−5^ M phenylephrine.

### Cell Culture

The embryonic rat aortic smooth muscle (A7r5) cell line was obtained from ATCC (Cat# CRL-1444, RRID:CVCL_0137). Since these cells are of embryonic origin as assumed to be a mixture of both male and female cells, additional experiments utilized primary aortic smooth muscle cells isolated from the thoracic aorta of male and female Sprague Dawley rats (12–14 weeks of age). Cells were cultured for up to ten passages in Media 199 containing 10% FBS, 1% penicillin-streptomycin and 1% L-glutamine in 95% air, 5% CO_2_ in 37°C incubator. Cells were grown to near confluence (80%-90%) then switched to phenol red-free Media 199 containing 0.5% charcoal-stripped serum, 1% penicillin-streptomycin, and 1% L-glutamine. Cells were treated in the presence or absence of GPER agonist G-1 (100 nM), GPER antagonist G36 (10 μM) (38), adenylyl cyclase inhibitor SQ22536 (5 μM), and phosphodiesterase-4 inhibitor rolipram (5 μM) for 24 h before being exposed to Ang II (100 nM) for 4 h.

### Immunoblotting

After treatment, cells were washed and collected in ice cold phosphate-buffered solution then lysed in RIPA buffer containing protease and phosphatase inhibitors. Protein was estimated using the Pierce™ BCA Protein Assay Kit, and 50 μg of protein was resolved in a 10% Sodium dodecyl sulfate gel by electrophoresis before being transferred to a 0.45 μm nitrocellulose membrane for 2 h. Membranes were blocked in 5% non-fat milk for 2 h and incubated overnight with anti-Nox4 (1:1,000; Abcam ab133303-16), an antibody whose protein specificity was validated in other studies (39) as well as in our hands using siRNA (**Figure 6D**). Blots were reprobed with anti-GAPDH (GeneTex gtx627408) or anti-β actin (Cell Signaling Technology 3700) as a loading control. Secondary antibodies against rabbit and mouse were used at a 1:1,000 dilution. Image Studio Lite Version 5.2 was used to analyze band intensity.

### Histology

Aortas and hearts were fixed overnight in 10% PBS buffered formalin and followed by storage in 70% ethanol. Paraffin-embedded sections were stained with hematoxylin and eosin, and aortic wall thickness was measured by taking measurements along the aortic wall circumference perpendicular to the lumen at 10 points per sample. To assess for cardiac hypertrophy, the entire cross-section of the heart was imaged at 4× magnification with the Cytation 5 imaging reader (BioTek, Winooski, VT). Wall and lumen areas were measured to calculate the LV/lumen ratio.

### RNA Extraction and Reverse Transcription-Quantitative PCR

Cells or tissues were subjected to RNA extraction using the Qiagen RNeasy mini kit (cat# 74106). The amount of RNA was estimated using a NanoDrop 3300 Fluorospectrometer (RRID:SCR_015804). For real-time polymerase chain reaction, a total of up to 500 ng RNA was used for PCR reaction. Specific rat primers for NOX4 (assay ID:qRnoCID0003969), NOX1 (assay ID:qRnoCID0004920), and GAPDH (assay ID:qRnoCID0057018) were obtained from Bio-Rad. For real-time PCR, iTaq™ Universal One-Step RT-qPCR Kit (cat# 172-5151) was used. The reaction mixture was set for 10 min at 50°C for cDNA synthesis, 5 min at 95°C for reverse transcription inactivation, and 10 s at 95°C for PCR cycling. Detection was done for 30 cycles followed by 30 s in 60°C and a melt curve analysis for 1 min at 95°C. The Bio-Rad® CFX96™ real-time PCR system was used to perform the assay in triplicate. To calculate the fold changes in mRNA expression, we normalized cycle threshold [C(t)] value of target genes to reference gene GAPDH using the 2^−ΔΔ*Ct*^ method.

### Nox4 siRNA Transfection

Small interference RNA duplexes targeting Nox4 (rat) were obtained from Origene (cat# SR506919). Cells seeded in 35 mm dishes were grown to 60% confluence, and 20 μl of transfection reagent Lipofectamine® Plus™ was added to 180 μl Opti-MEM™ media for a final volume of 200 μl. One hundred nanomolar of siRNA was then added and mixed in a 1 ml tube and left to stand for 20 min at room temperature. The reaction mixture was added to each well to a final volume of 4 ml Opti-MEM™ media. Cells were grown for an additional 30 h after transfection before experiments were performed.

### NADP/NADPH-Glo™ Assay

Cells treated in 24-well plates were washed with 37°C PBS and left to equilibrate at room temperature. NADP/NADPH-Glo™ detection reagent (cat#G9081, Promega) was added to each well and placed on a shaker for 1 h at room temperature. Luminescence was recorded using a Synergy™ HTX Multi-Mode Microplate Reader and normalized to protein in each well and expressed as relative luminescence units (RLU) per mg protein.

### Catalase Colorimetric Assay Activity

Cells cultured in 12-well plates were washed twice in ice cold PBS and scraped followed by centrifugation at 250 × g for 10 min. The cell pellet was collected and re-suspended in 1X assay buffer and sonicated. Finally the lysate was centrifuged at 10,000 × g for 15 min and a portion of the supernatant was subjected to catalase colorimetric activity kit (ThermoFisher Scientific cat# EIACATC). Briefly, the generated standard curve and protein absorbance was read at 560 nm using Synergy™ HTX Multi-Mode Microplate Reader and normalized to protein in each well-estimated by BCA method. Final results are expressed as units (U) per mg protein.

### Electron Spin Resonance Spectroscopy (ESR)

ESR was used to measure ROS in cells and isolated aortic tissues using the spin probe 1-hydroxy-3-methoxycarbonyl-2, 2, 5, 5-tetramethyl-pyrrolidine (CMH) as previously described (40). Diethyldithiocarbamate (DETC; 2.5 μmol/l) and desferoxamine (DF, 25 μmol/l) were dissolved under nitrogen gas bubbling in ice-cold modified Krebs-Hepes (KH) buffer. Media containing drug treatments was removed before analysis, to avoid potential interference with the CMH signal. Cells or tissues were washed with calcium- and magnesium-free Dulbecco's phosphate-buffered saline (DPBS) and incubated with freshly prepared CMH (200 μmol/L) solution in KH buffer containing DETC and DF at 37°C for 60 min. Samples with buffer were transferred to 1 ml syringes, snap frozen in liquid nitrogen, and stored at −80°C until analysis. Samples were transferred to a finger Dewar vessel (Noxygen Science Transfer and Diagnostics, Germany) and analyzed using an EMX ESR Benchtop spectrometer (Bruker, Germany) with the following ESR settings: center field, 1.99 g; microwave power, 20 mW; modulation amplitude, 2 G; sweep time, 10 s; number of scans, 10; field sweep, 60 G. The amplitudes of the spectra were normalized using protein concentration and expressed as arbitrary units per mg protein.

### Statistics

Statistical analysis was performed using GraphPad Prism 6.07 software (GraphPad Software). Outliers were identified using the ROUT method. For one factor analysis, the Shapiro-Wilk test was used to determine normality. Unpaired *t*-test was used to determine the difference between two groups. One-way ANOVA was used to determine differences between three or more groups, and if significant Tukey's multiple comparison test was performed. For data that was not normally distributed, Kruskal-Wallis with Dunn's multiple comparison was used. Two-way repeated measures ANOVA was used to analyze timeline and sex differences data, with no assumptions of sphericity, Geisser-Greenhouse corrections, and Tukey's test. For pre/post data, sphericity was assumed and multiple comparisons were made with Sidak's test. Comparisons where *P* < 0.05 were considered significant. All experiments were repeated at least once. Information on statistical tests used are also provided in graph legends.

## Results

### GPER Deletion in Females Impacts Pulse Pressure but Not Mean Arterial Pressure

**Figure 1:**
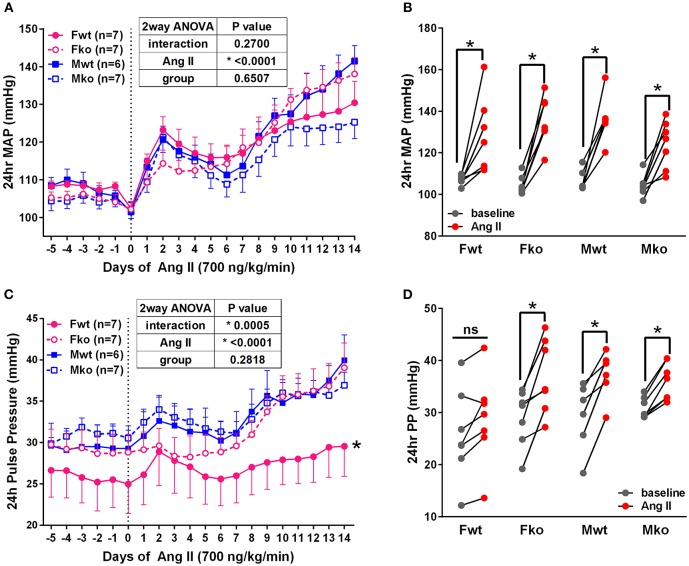
GPER deletion increases pulse pressure in hypertensive female mice. **(A)** Radiotelemetry recordings show that MAP is not different at baseline or during 2-week Ang II infusion. **(B)** Grouped MAP pre and post Ang II shows a significant increase in all groups. 2-way repeated measures ANOVA, *p* < 0.0001 for Ang II, Sidak, ^*^p < 0.005 for all groups. **(C)** Pulse pressure was significantly lower in Fwt mice. Tukey's test for main column effect, ^*^p < 0.001 vs. all other groups. **(D)** Grouped pulse pressure data pre and post Ang II shows a significant increase in all groups except Fwt. 2-way repeated measures ANOVA, *p* < 0.0001 for Ang II, Sidak, ^*^p < 0.005 vs. baseline.

To determine the impact of genetic GPER deletion on cardiovascular parameters at baseline and during hypertension, male and female (M and F) wildtype and GPER knockout (wt and ko) mice were implanted with telemetry probes and exposed to Ang II for 2 weeks. As shown in Figure 1, no significant differences in MAP were found at baseline or in response to Ang II (Figure 1A). There was a trend for lower MAP in Mko mice that did not reach statistical significance. Ang II induced a significant increase in blood pressure in all groups (Figure 1B). Analysis of systolic and diastolic pressures did not reveal any impact of genotype (Table 1). The ability of Ang II-induced hypertension to decrease heart rate was significant only in the Mwt group. Interestingly, Fwt mice had significantly lower pulse pressures than all other groups, while Fko pulse pressure was similar to Mwt and Mko mice (Figure 1C). In addition, pulse pressure was significantly increased by Ang II hypertension in all groups except Fwt mice (Figure 1D).

**Table 1:** Cardiovascular parameters.

	**Fwt**	**Fko**	**Mwt**	**Mko**	**2-way ANOVA**		
					**Interaction**	**Ang II**	**Group**
Baseline SBP	120 ± 2	118 ± 2	121 ± 2	119 ± 2	0.3337	* <0.0001	0.2670
Ang II SBP	142 ± 7	151 ± 6	156 ± 4	142 ± 5			
Baseline DBP	94 ± 2	89 ± 1	92 ± 2	88 ± 2	0.4870	* <0.0001	0.3790
Ang II DBP	114 ± 7	115 ± 4	118 ± 6	106 ± 4			
Baseline HR	596 ± 16	595 ± 10	550 ± 8	571 ± 12	0.4966	*0.0024	*0.0013 (Sidak, Mwt, *P* = 0.04)
Ang II HR	588 ± 13	580 ± 8	517 ± 11	551 ± 9			

*Mean ± SEM and statistical analysis of systolic blood pressure (SBP, mmHg), diastolic blood pressure (DBP, mmHg), and heart rate (HR, beats-per-minute) obtained from 24 h telemetry recordings at baseline and after 2-week infusion of Ang II*.

### Impact of GPER Deletion on Wall Thickness, Nox Expression, and Vascular Reactivity

**Figure 2:**
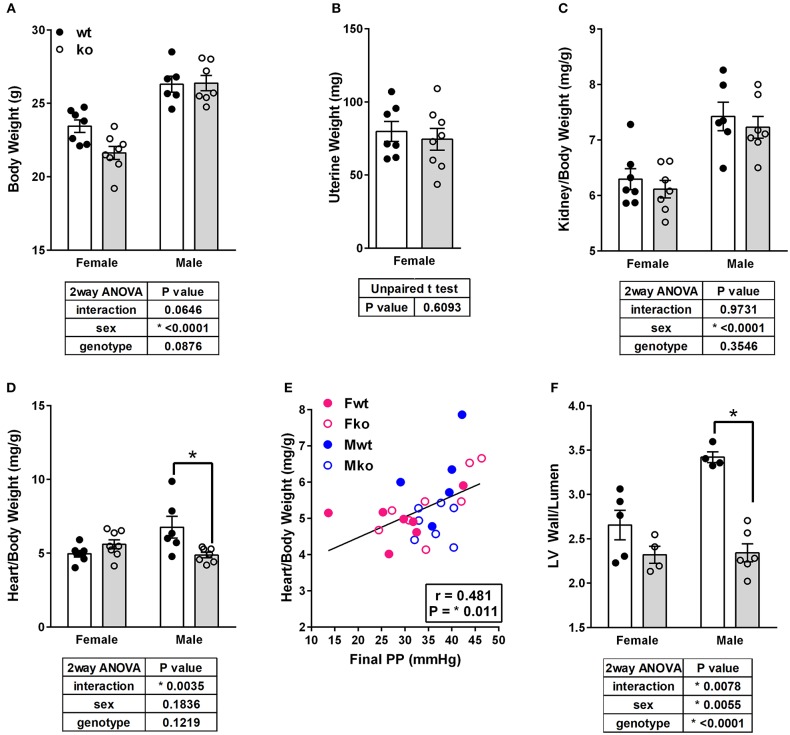
GPER deletion impacts tissue weights and left ventricle wall thickness. **(A)** Body weight, **(B)** Uterine weight, **(C)** Kidney-to-body weight ratio, and **(D)** Heart-to-body weight ratio, all with respective statistical test results and Sidak's test if applicable. **(E)** A significant and positive correlation was found between final pulse pressure and heart-to-body weight ratio. **(F)** Left ventricular (LV) wall thickness-to-lumen ratio was higher in Ang II-infused Mwt versus Mko mice.

**Figure 3:**
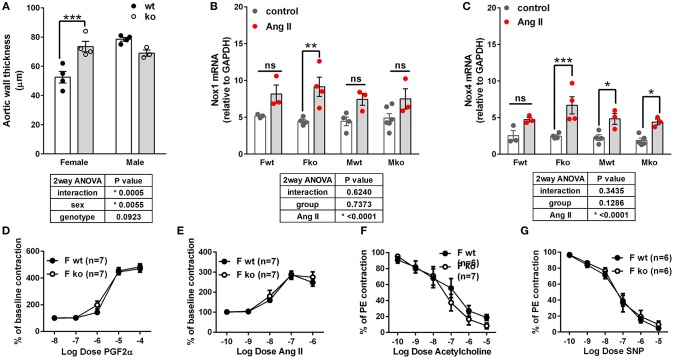
Impact of GPER deletion on aortic wall thickness, Nox expression and vascular response. **(A)** Aortic wall thickness was increased in Ang II-infused Fko vs. Fwt mice, Sidak, ^***^p < 0.001. **(B)** Nox1 mRNA was significantly increased by Ang II only in Fko mice, Sidak, ^**^p < 0.005. **(C)** Nox4 mRNA was significantly increased by Ang II in all groups except Fwt, Sidak, ^*^p < 0.05, ^***^p < 0.001. Reactivity of Fwt and Fko mesenteric arteries after Ang II-infusion were not different when comparing **(D)** PGF2α-mediated contraction, **(E)** angiotensin (Ang) II-mediated contraction, **(F)** acetylcholine-induced vasorelaxation, and **(G)** sodium nitroprusside (SNP)-mediated relaxation.

In Ang II-infused mice, body weight and kidney weight ratios were significantly higher in males but not impacted by genotype (Figures 2A,C). Uterine weights were not impacted by genotype (Figure 2B). A sex difference in cardiac weight ratios was observed in wt but not GPER ko mice (Figure 2D). Left ventricular wall-to-lumen ratio was impacted by GPER ko in male but not female mice (Figure 2F). A significant and positive correlation was found between final pulse pressure and heart-to-body weight ratio (Figure 2E). Assessment of aortic cross sections indicated a significant interaction between genotype and sex with increased wall thickness in Fko vs. Fwt with no impact in male mice (Figure 3A). To investigate the impact of Ang II on aortic Nox4 and Nox1, a separate cohort of mice was infused with Ang II for 2 weeks at the same dose or used as controls. Nox4 mRNA was significantly increased by Ang II treatment in all groups except Fwt mice (Figure 3B), while Nox1 mRNA was increased in female ko mice only (Figure 3C). Mesenteric arteries from female GPER ko and wt mice infused with Ang II were also assessed for vascular reactivity. Vessel contraction to increasing concentrations of PGF2α or Ang II was not significantly different between Fko and Fwt mice (Figures 3D,E). Similarly, relaxation to acetylcholine or SNP in pre-constricted vessels was also comparable between GPER ko and wt females (Figures 3F,G).

### GPER Activation Prevented Ang II-Induced Increases in ROS and NADP/NADPH Ratio

**Figure 4:**
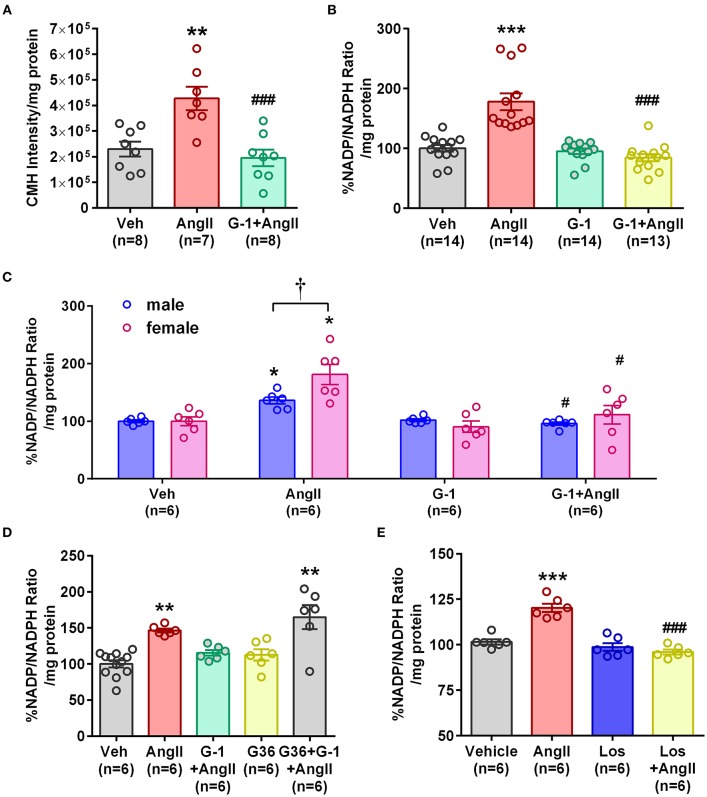
GPER activation prevented Ang II-induced increases in ROS and NADP/NADPH ratio. **(A)** Stimulation of A7r5 cells with 100 nM Ang II for 4 h significantly increased ROS, which was prevented by pretreatment with the GPER agonist G-1 for 24 h. 1-way ANOVA, *p* = 0.0004, Tukey ^**^p < 0.01 vs. vehicle (Veh); ^###^*p* < 0.0001 vs. Ang II. **(B)** Ang II-induced increases in the NADP/NADPH ratio were also mitigated by G-1. Kruskai-Wallis test, *p* < 0.0001, Dunn, ^***^p < 0.001 vs. Veh; ^###^*p* < 0.001 vs. Ang II. **(C)** Ang II also increased NADP/NADPH ratio in primary male and female Sprague Dawley rat aortic smooth muscle cells, which was reversed by G-1. 2-way ANOVA with Geisser-Greenhouse correction, ^*^p < 0.0323 for interaction, Tukey, ^*^p < 0.05 vs. Veh; ^#^*p* < 0.05 vs. Ang II; ^†^p < 0.01 sex effect. **(D)** The GPER antagonist G36 blocked the ability of G-1 to inhibit Ang II effects on NADP/NADPH ratio. 1-way ANOVA, *p* < 0.0001, Tukey, ^**^p < 0.01 vs. Veh. **(E)** The increase in NADP/NADPH ratio induced by Ang II was completely prevented by losartan (Los). 1-way ANOVA, *p* < 0.0001, Tukey, ^***^p < 0.001 vs. vehicle; ^###^*p* < 0.001 vs. Ang II.

To determine whether GPER impacts vascular Nox4 and oxidative stress, we designed *in vitro* experiments using A7r5 aortic smooth muscle cells (ASMC). Ang II-induced ROS was prevented by the GPER agonist G-1 (Figure 4A). Similarly, G-1 blocked Ang II-induced increases in NADP/NADPH ratio but did not alter levels when given alone (Figure 4B). Since A7r5 cells are embryonic and most likely contain a mixture of male and female cells, we assessed sex differences in the impact of GPER on oxidative stress. Surprisingly, primary isolated female ASMC had higher levels of ROS when assessed in estrogen-free conditions, but G-1 similarly attenuated the impact of Ang II in cells from both sexes (Figure 4C). To confirm involvement of GPER, cells were treated with Ang II and G-1 in the presence or absence of the GPER antagonist G36. The data consistently showed that Ang II increased while G-1 reversed NADP/NADPH ratio, but blocking GPER with G36 rendered G-1 ineffective in reversing the effect of Ang II (Figure 4D). To confirm the role of the Ang II type 1 receptor (AT1R) in mediating the effects of Ang II, we examined NADP/NADPH ratio in the presence or absence of the AT1R antagonist losartan (Figure 4E). Losartan completely blocked the Ang II-induced increase in NADP/NADPH ratio.

### GPER and Ang II Regulate Nox4 Protein and mRNA

**Figure 5:**
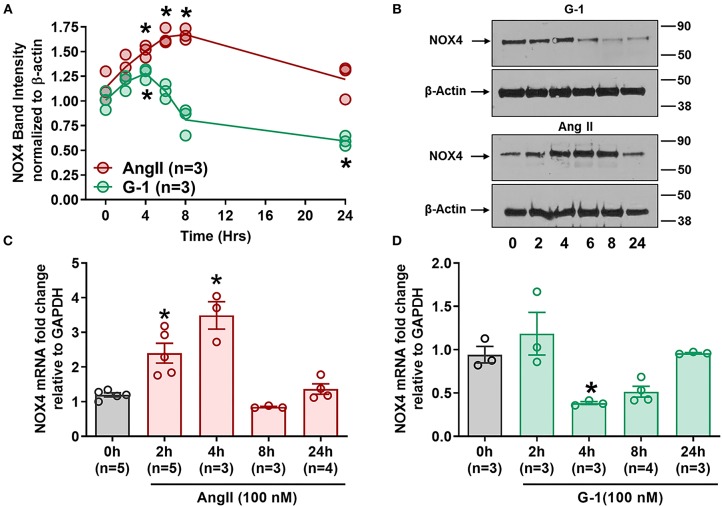
GPER and Ang II regulate Nox4 protein and mRNA. **(A)** Ang II increased Nox4 protein at 4, 6, and 8 h while G-1 downregulated Nox4 protein at 24 h. 2-way ANOVA, *p* < 0.001 for interaction, Dunnett, ^*^p < 0.05 vs. 0 h. **(B)** Representative blots for **(A)**. **(C)** Ang II and **(D)** G-1 also impacted Nox4 mRNA. 1-way ANOVA, *p* < 0.01, Dunnett, ^*^p < 0.05 vs. 0 h.

Since GPER attenuated Ang II-induced ROS and NADP/NADPH ratio, we next determined its impact on Nox4. Ang II upregulated Nox4 protein in A7r5 cells at 4, 6, and 8 h compared with baseline, while G-1 significantly decreased Nox4 protein expression at 24 h (Figures 5A,B). RT-qPCR showed that Nox4 mRNA levels were significantly increased by Ang II at 2 and 4 h (Figure 5C) but were decreased by G-1 at 4 h when compared with controls (Figure 5D). Nox4 mRNA was restored to control levels after 24 h of either G-1 or Ang II. These experiments indicated that Ang II and GPER regulate Nox4 in opposite directions at the transcriptional level.

### GPER Activation Prevents Ang II-Induced Upregulation of Nox4 Protein

**Figure 6:**
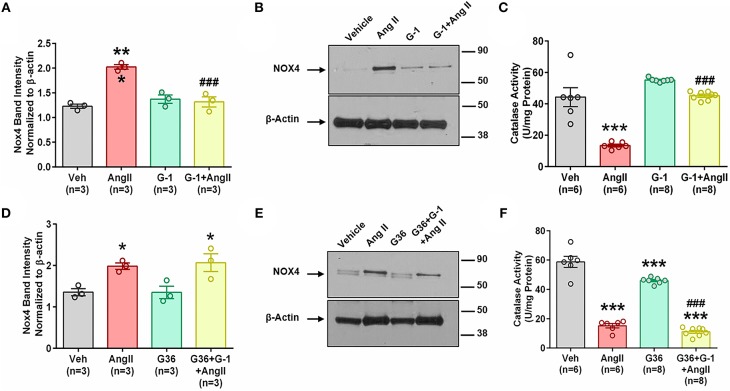
GPER activation prevents Ang II-induced upregulation of Nox4 protein. **(A)** Ang II-induced Nox4 protein is reversed by G-1. 1-way ANOVA, *p* = 0.0002, Tukey, ^***^p < 0.001 vs. vehicle (Veh); ^###^*p* < 0.001 vs. Ang II. **(B)** Representative blot for **(A)**. **(C)** Ang II-induced decreased catalase activity while G-1 prevented the decrease. 1-way ANOVA, *p* < 0.0001, Tukey, ^***^p < 0.001 vs. vehicle; ^###^*p* < 0.001 vs. Ang II. **(D)** The GPER antagonist G36 blocked the ability of G-1 to inhibit Ang II effects on Nox4 protein. 1-way ANOVA, *p* = 0.0106, Tukey, ^*^p < 0.05 vs. Veh. **(E)** Representative immunoblot for **(C)**. **(F)** G36 blocked the effect of G-1 on Ang II-induced decreased catalase activity. 1-way ANOVA, *p* < 0.0001, Tukey, ^***^p < 0.001 vs. Veh; ^###^*p* < 0.001 vs. G36.

We next determined whether pretreatment with the GPER agonist prevented Ang II-induced increases in Nox4 mRNA and protein. G-1 pretreatment for 24 h did not alter Nox4 expression alone but prevented the upregulation induced by Ang II (Figures 6A,B). We used catalase activity to indirectly determine the amount of H_2_O_2_ produced when we blocked or activated GPER. Ang II significantly downregulated catalase activity, but this effect was ameliorated in the presence of G-1 (Figure 6C). The antagonist G36 eliminated the ability of G-1 to inhibit the effects of Ang II on Nox4 protein (Figures 6D,E) as well as catalase activity (Figure 6F).

### siRNA Knockdown of Nox4 Reduced Ang II Effects

**Figure 7:**
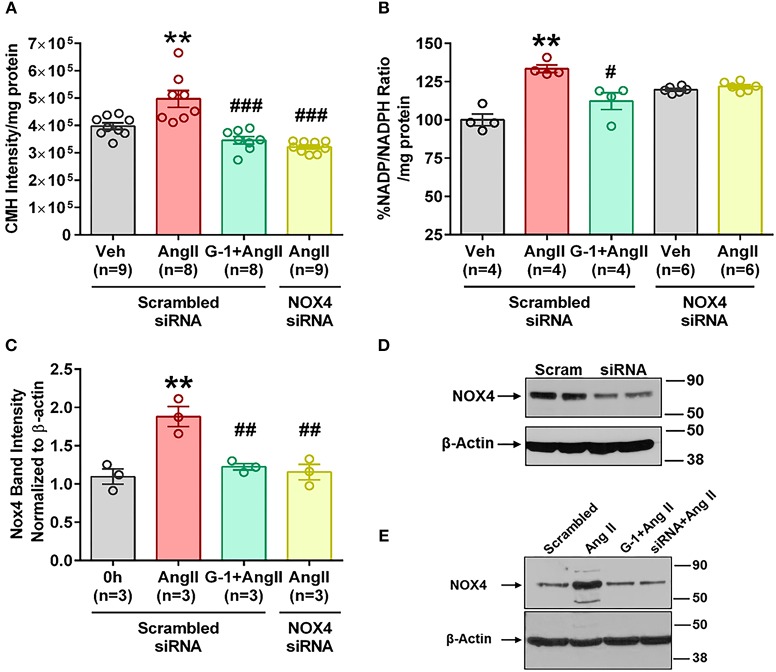
siRNA knockdown of Nox4 reduced Ang II effects. **(A)** Nox4 siRNA significantly reduced Ang II-induced ROS. 1-way ANOVA, *p* < 0.0001, Holm-Sidak, ^**^p < 0.01 vs. Veh; ^###^*p* < 0.0001 vs. Ang II. **(B)** Nox4 siRNA significantly reduced Ang II-induced NADP/NADPH ratio. Kruskai-Wallis test, *p* = 0.0012, Dunn ^**^p < 0.01 vs. Veh; ^#^*p* < 0.05 vs. Ang II. **(C)** Ang II upregulated Nox4 protein in the presence of scrambled (Scr) siRNA but not with Nox4 siRNA. 1-way ANOVA, *p* = 0.0017, Tukey, ^*^p < 0.05 vs. Veh; ^##^*p* < 0.01 vs. Ang II. **(D)** Validation of Nox4 antibody and siRNA. **(E)** Representative blot for **(C)**.

We utilized small interference (si) RNA for Nox4 to determine the role of this protein in Ang II-mediated oxidative stress, and validation of protein downregulation is shown in Figure 7D. Nox4 siRNA completely abrogated Ang II-induced ROS production (Figure 7A) and NADP/NADPH ratio (Figure 7B) compared with scrambled siRNA controls. Nox4 knockdown also prevented Ang II-induced Nox4 protein upregulation by 80% (Figures 7C,E), an effect similar to that seen with G-1 treatment.

### Impact of cAMP Signaling

**Figure 8:**
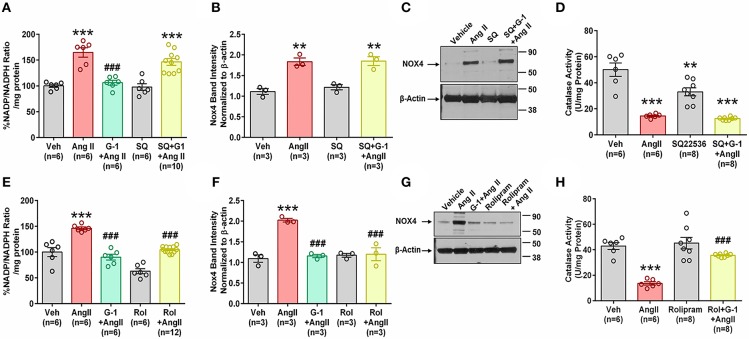
Impact of cAMP signaling. The adenylyl cyclase inhibitor SQ22536 (SQ) blocked the ability of GPER to oppose the impact of Ang II on **(A)** NADP/NADPH ratio. 1-way ANOVA, *p* < 0.0001, Tukey, ^***^p < 0.001 vs. Veh, ^###^*p* < 0.001 vs. Ang II. **(B)** Nox4 protein. 1-way ANOVA, *p* = 0.0003, Holm-Sidak, ^**^p < 0.01 vs. Veh. **(C)** Representative blot for **(B)**. **(D)** SQ also blocks G-1's ability to reverse Ang II-induced decreased catalase activity. 1-way ANOVA, *p* < 0.0001, Tukey, ^**^p < 0.01, ^***^p < 0.001 vs. Veh. Increasing cAMP with the phosphodiesterase inhibitor rolipram (Rol) mimics the impact of G-1 on **(E)** NADP/NADPH ratio. 1-way ANOVA, *p* < 0.0001, Tukey, ^***^p < 0.001 vs. Veh, ^###^*p* < 0.001 vs. Ang II. **(F)** Nox4 protein. 1-way ANOVA, *p* < 0.0001, Tukey, ^###^*p* < 0.001 vs. Veh, ^###^*p* < 0.001 vs. Ang II. **(G)** Representative blot for **(E)**. **(H)** Ang II-induced decreased catalase activity is restored by Roland G-1. 1-way ANOVA, *p* < 0.0001, Tukey, ^***^p < 0.001 vs. Veh; ^###^*p* < 0.001 vs. Ang II.

We next investigated the role of GPER-mediated cAMP production in the protective effects of G-1 on NADPH oxidase activity and Nox4 protein expression. A7r5 cells were treated with Ang II in the presence or absence of the GPER agonist G-1, the adenylyl cyclase inhibitor SQ22536 (SQ), or the phosphodiesterase (PDE) 4 inhibitor rolipram, which increases intracellular cAMP levels by preventing its breakdown. G-1 again prevented Ang II-induced increases in NADP/NADPH ratio, but SQ blocked this effect (Figure 8A). Similarly, when adenylyl cyclase was inhibited, G-1 was unable to prevent the effect of Ang II on Nox4 protein (Figures 8B,C) and catalase activity (Figure 8D). Rolipram mimicked the effects of GPER activation by blocking Ang II-induced NADP/NADPH activity (Figure 8E), Nox4 upregulation (Figures 8F,G), and decreased catalase activity (Figure 8H).

## Discussion

The novel finding from the current study is that despite similar blood pressures, GPER deletion in female mice significantly increased pulse pressure and exacerbated the upregulation of aortic NADPH oxidases in response to Ang II. In parallel, *in vitro* activation of GPER attenuated oxidative stress via cAMP-mediated regulation of Nox4. We demonstrated that Nox4 plays a major role in Ang II-induced ROS production in cultured VSMC. Furthermore, we showed that GPER opposed the effects of Ang II by downregulating Nox4 at the transcriptional level and restoring catalase activity. These findings have important implications since randomized clinical trials fail to significantly inhibit oxidative stress using currently available antioxidants such as vitamin E (41–43), while preclinical studies with Nox1 and 4 inhibitors (44, 45) are promising. Since oxidative stress is detrimental to cardiovascular tissues, GPER may provide a novel target for inhibition of vascular ROS.

In contrast to previous studies, we did not find a sex difference in Ang II-induced hypertension as found in other studies using the same or similar protocol (36, 37). A study using a higher dose of Ang II also failed to detect sex differences in telemetry blood pressure recordings (46). Since the mice used in this study were developed from a 129/Sv strain and backcrossed to the C57bl/6, some genetic aspects of the 129/Sv strain may have carried over (34, 47), since mice of this strain have two renin genes and higher blood pressures even after two generations of backcrossing with C57bl/6 mice (48, 49). Four GPER knockout mice strains have been created using slightly different methods (13), and in this study the model created by homologous recombination was utilized (17, 34). The results from these different models is varied, for example previous data from this same strain utilized in the current study shows increased body weight in both male and female knockout mice at 10 months of age (15). In contrast, data from a different GPER knockout strain shows lower body weight in Fko at 19 weeks of age, and also finds increased mean arterial pressure in female knockout mice at 9 months of age but not at 6 months, the latter of which is comparable to the ages of the mice in our study (17). We found no difference in body weight or blood pressure before or after Ang II infusion, suggesting that age and strain are important factors in observing these phenotypes.

Since sex did not influence Ang II hypertension and the hypothesis was that GPER deletion would remove sex differences, we were not surprised to find similar MAP in wt and ko mice. However, we found a significant impact of GPER deletion when analyzing pulse pressure, an indicator of arterial stiffening, which was increased in Fko mice to a level comparable to male wt and ko mice. These data support our previous study in salt-loaded mRen2 rats where despite similar levels of hypertension, aortic wall thickness was significantly reduced by chronic treatment with the GPER agonist G-1 (9). The increased stiffness observed may precede changes in pressure, considering that arterial stiffening is observed before increases in blood pressure in aging humans as well as a mouse model of high fat diet-induced hypertension (50–52). While arterial stiffening increases afterload, cardiac hypertrophy was not different in Fwt vs. Fko mice in the current study. However, the significant positive correlation with pulse pressure indicates that arterial stiffening is associated with increased cardiac remodeling, but longer Ang II infusion may be required to observe differences between groups. The current study indicates that in female mice, GPER provides protection from Ang II-induced vascular remodeling and pulse pressure increases, but not hypertension.

Using ESR spectroscopy, the best method for detecting and analyzing ROS in biological samples (40), we confirmed that GPER activation promotes antioxidant defenses in the vasculature. This result is consistent with studies showing that estrogen attenuates oxidative stress in VSMC (53) and endothelial cells (6). Previous work from our laboratory indicates that GPER attenuates vascular oxidative stress and remodeling (9), decreases cardiac ROS (11), and reduces renal oxidative damage in rats fed a high salt diet (10). In addition, deficiency of GPER in cardiomyocytes of female mice is associated with increased cardiac oxidative stress (33). Mechanisms for the antioxidant effect of GPER in other cell types include a reduction in mitochondrial permeability transition pore opening in cardiac cells (54), regulation of several antioxidant genes including glutathione peroxidase and thioredoxin-interacting protein in skeletal muscle (33), and reduced H_2_O_2_ peroxidation in the liver (6). Recent evidence also suggests that GPER induces superoxide dismutase during methotrexate-induced kidney damage (55). In the rat heart, ovariectomy increases Nox4 expression and oxidative stress and is reversed by administration of the GPER agonist G1 (11). Notably, deletion of GPER in cardiomyocytes increases Nox4 and oxidative stress in female mice (33), suggesting a detrimental role for this enzyme in the heart (56). These studies indicate a connection between GPER, oxidative stress, and Nox4 in multiple cardiovascular tissues.

Our experiments using GPER ko and wt mice indicate that females with intact GPER signaling were protected from Ang II-induced increases in aortic Nox1 and Nox4. While the ability of Ang II to upregulate Nox1/2 and negatively impact cardiovascular health is well-established, the role of Nox4 is still debatable. Studies show both up and downregulation of Nox4 in response to Ang II (30, 57–60). Functionally, Nox4 induces VSMC hypertrophy, oxidation of lipids, and inactivation of nitric oxide (23, 29), but is required for VSMC differentiation (61) and protects endothelial cells during hypoxia (62). This conflicting data is also observed in Nox4 knockout mice, where Ang II-hypertension is not impacted but aortic wall thickness is *increased* (63). Nox4-induced endothelium derived hyperpolarizing factor mediates a decrease in blood pressure, suggesting a vasculoprotective role (64). While Nox4 promotes nitric oxide production during shear stress in endothelial cells, during aging Nox4 uncouples endothelial nitric oxide synthase and induces oxidative stress (65). Importantly for the increased pulse pressure and aortic remodeling observed in the current study, Nox4 is upregulated in aortic smooth muscle during aging and contributes to mitochondrial ROS and vascular stiffening (66). The same group recently showed that overexpression of mitochondrial Nox4 increases aortic smooth muscle cell stiffness and pulse wave velocity (67). Similarly, pharmacological inhibition of Nox4 using GKT137831 attenuates hypoxia-induced pulmonary artery remodeling (68), and a Nox4 dominant negative mutation protects atherosclerotic mice from increases in pulse wave velocity (69). Since GPER antagonism reduces aortic ROS in aged male mice and was associated with downregulation of Nox1 but not Nox4 (21), the role of Nox4 in the vasculature may be altered during the aging process.

Another factor may be the relative amounts of superoxide vs. H_2_O_2_ that are produced by the Nox4 enzyme, which may depend on cell type. Nox4 produces superoxide in neurons and rat aortic smooth muscle cells (31, 70), but H_2_O_2_ in endothelial cells (71). The protective vs. detrimental impact of H_2_O_2_ may also differ by cell type. In a tamoxifen-inducible endothelial Nox4 knockout mouse model, H_2_O_2_ produced by Nox4 increases angiogenesis after femoral artery ligation injury demonstrating a protective role (63). However, smooth muscle cell overexpression of catalase, which quenches H_2_O_2_, protects from Ang II-induced aortic remodeling (23). Our data indicates that in cultured vascular smooth muscle cells, catalase activity is reduced in the presence of Ang II and reversed by the GPER agonist G-1. Ang II most likely downregulates catalase activity by increasing superoxide which reacts with superoxide dismutase to form H_2_O_2_ (72).

Since most studies are performed only in male mice, sex differences in Nox4 expression or function may underlie its beneficial vs. detrimental vascular effects. In males, Nox4 is highly expressed in basilar cerebral arteries (73), while females express high Nox4 in mesenteric (74) and porcine coronary arteries (75), suggesting sex and functional differences. Sexual dimorphisms may also become important when considering GPER, since we found a trend for lower MAP and cardiac protection in Mko mice in the current study. Similarly, GPER antagonism in aging male mice confers protection from oxidative stress by decreasing vascular Nox1 with no impact on Nox4 (21). Nox1 and 4 may interact in the regulation of ROS since non-specific Nox1/4 inhibitors GKT136901 or GKT137831 attenuate oxidative stress (44, 45, 68). Our study indicates that in female mice infused with Ang II, Nox4 plays a detrimental role in vascular smooth muscle cell remodeling, while intact signaling by GPER confers protection.

Surprisingly, activation of a G protein-coupled receptor known for its role in acute estrogen signaling had a significant impact on Nox4 mRNA within 4 h. Other GPCRs such as endothelin-1 and thrombin receptors also regulate NADPH oxidases (76). Our study also demonstrated that cAMP activation by GPER is necessary to regulate both NADP/NADPH ratio and Nox4 expression. Activation of GPER by the agonist G-1 activates adenylyl cyclase, leading to accumulation of cAMP (77), and work from our lab and others show that this GPER signaling cascade induces vasorelaxation (8, 78). Downstream phosphorylation of protein kinase A activates cAMP response element binding protein (CREB), a transcription factor that regulates several genes including Nox1 and Nox5 (79, 80). Activation of the cAMP-CREB pathway attenuates VSMC migration (81), while reductions in cAMP increase NADP oxidation to promote ROS in aortic smooth muscle cells (31, 82). Our results connecting GPER-induced cAMP increases with Nox4 regulation and NADP/NADPH ratio indicate that this signaling pathway plays an important role in attenuating Ang II-induced oxidative stress and remodeling. Interestingly, Ang II-induced downregulation of catalase activity can also be reversed by G-1 and rolipram, suggesting a distinct pathway involving the stimulation of adenylyl cyclase and accumulation of cAMP for protection against Ang II-induced ROS.

In addition to the current findings, activation of GPER attenuates oxidative damage to pancreatic beta cells in diabetes (47) and protects neurons from oxidative stress in the brain (83). Since oxidative stress is involved in many disease processes and GPER is ubiquitously distributed in mammals, therapeutic targeting of this estrogen receptor may provide benefits. While menopause is associated with an increase in cardiovascular disease, vascular stiffening, and increased ROS production (84), GPER may have the capacity to selectively decrease oxidative stress without activating nuclear estrogenic signaling. Therefore, inclusion of GPER as a therapeutic target may alleviate deleterious effects in both cardiovascular and metabolic diseases.

## Data Availability

The datasets generated during the current study are available in the Harvard Dataverse repository: https://dataverse.harvard.edu/dataset.xhtml?persistentId=doi:10.7910/DVN/Z9FEPX.

## Ethics Statement

All procedures were carried out in accordance with the NIH Guide for the Care and Use of Laboratory Animals and approved by the Tulane University Institutional Animal Care and Use Committee.

## Author Contributions

BO, KM, PK, and SL contributed conception and design of the study. BO, VS, JD, KG, MZ, and GC performed the experiments. BO and SL performed the statistical analysis. BO wrote the first draft of the manuscript. SL wrote sections of the manuscript. All authors contributed to manuscript revisions and approved the submitted version.

### Conflict of Interest Statement

The authors declare that the research was conducted in the absence of any commercial or financial relationships that could be construed as a potential conflict of interest.
